# Tropho-Neurosis of the Oral Cavity with Especial Reference to Syphilitic Necrosis

**Published:** 1890-09

**Authors:** G. Frank Lydston

**Affiliations:** Chicago, Ill.


### THE DENTAL REGISTER.

VOL. XLIV.] SEPTEMBER, 1890. [No. 9.

### COMMUNICATIONS.

### Tropho-Neurosis of the Oral Cavity with Especial Reference to Syphilitic Necrosis.

BY G. FRANK LYDSTON, M.D., OF CHICAGO, ILL.

Read in the Section of Dental and Oral Surgery at the Forty-first Annual Meeting of the American Medical Association at Nashville, Tenn., May 22, 1890.

At the meeting of the Southern Surgical and Gynæcological Association held in Nashville, November, 1889, I had the pleasure of presenting certain views regarding the relation of tropho-neurosis to the phenomena of syphilis. In that essay I endeavored to show the dependence of every phenomenon characteristic of syphilis upon certain more or less obscure changes in the sympathetic nervous system (organic or functional, temporary or permanent), which resulted in aberations of its trophic function. The more thoroughly I studied the phenomena of syphilis, the more firmly convinced I became not only of the dependence of syphilitic phenomena, but that of many other morbid changes which have hitherto appeared inexplicable, upon tropho-neuroses.

In a general way it may be said that disease affecting the animal body can only be produced by one of two things, viz: (1) Perversion in the quality or quantity of the nutritive material which constitutes the pabulum upon which the various structures of the body feed; (2) some perversion of that nervous energy upon which building up of the tissues depends. As in building a house, if the workman be skilled and his materials good, the result is a good house. So in the building up of the tissues; if the nutrient pabulum (or lumber) be first-class, and the workman (the nervous system) performs his functions properly, the result will be healthy tissue. Now, what is it that acts as a governor for the process of tissue building? Certainly the sympathetic nervous system. The sympathetic system, through the medium of its trophic fibres, indubitably presides over the functions of nutrition, of waste and repair. Incidental to repair we have a reproduction of cell growth through the medium of the leucocytes. If the integrity of the sympathetic ganglia or their efferent nerves be impaired, the young cells in the process of growth, instead of developing into a normal deposit of connective tissue cells, fail to become perfectly differentiated; as a consequence they retain the physical characters of young or embryonal cells. Not only do these young cells fail to be transformed into vigorous tissue structure, but they manifest the usual tendency of embryonal cells, viz.: to rapidly proliferate and speedily degenerate.

It is found on section of the syphilides, wherever found, that they are composed of cells in no wise different from young connective tissue cells. Fessenden Otis lays much stress upon this point. There seems to be but little difference between the syphilized cell and that progenitor of all connective tissue cells—the leucocyte. An increased rapidity of proliferation, perhaps a trifling increase in size upon the average, and the power of infecting healthy tissue, are the only new properties which the cell acquires by virtue of its syphilization. With the exception of the property of infectiousness, these qualities are possessed by all imperfectly differentiated cells. Place a young connective tissue cell and a syphilitic cell under the microscope, and we can not tell the one from the other.

The morbid propensities of embryonal cells are well shown in cancer, in which disease, if Cohnheim be correct, they are the foundation of the morbid process. I firmly believe that malignant growths are explicable upon the same grounds as syphilitic neoplasia. It has seemed to me that the reason an injury of the tissues in one individual is followed by cancer, while in others normal repair occurs, is that in the former the functions of the sympathetic system are impaired, and instead of a normal differentiation and building up of the cells, there occurs a heaping up of embryonal cells. There is practically no difference between such embryonal cells and those which, according to Cohnheim, remain imprisoned in the fœtal structures to develop under proper stimulus into cancer in after life. The development of imprisoned embryonal cells this celebrated pathologist believes to be the *fons origo et mali* of cancer.

In considering abnormal tissue growth, we must first consider the medium through which normal tissue growth occurs. If we accept the view that normal tissue repair depends for its performance upon the integrity and function of the sympathetic nervous system, we must *nolens volens* accept the view that any pathological process in which a heaping up of tissue, degraded or otherwise, occurs, is produced through the medium of some aberration of the trophic function of the sympathetic. This, it seems to me, is an indisputable fact, and is the basis of my theory of the tropho-neurotic character of the phenomena of syphilis. There are numerous trophic disorders which are pertinent to the question of the dependence of the lesions of syphilis on a sympathetic neurosis; for example, bed sores, perforating ulcers incidental to paralysis, certain abscesses, changes in the bones and joints in spinal arthropathy or Charcot's joint disease, and gangræna vaso-motoria. Raynaud's disease, a peculiar nervous affection, is attended by gangrene of localized areas of the skin and certain portions of the extremities, which are unquestionably due to nervous disturbance. This disease was long confounded with erysipelas. Its phenomena are those of ischæmia of tissue, followed by erythema, cyanosis and gangrene.

I have noticed in syphilis, in certain instances, a marked tendency to fluctuations of vaso-motor impulse. Thus I have observed a number of cases in which there was a marked tendency to epistaxis, hæmoptysis, hæmorrhage from the bowels and kidneys. In several cases complicated by stricture I have observed a tendency to hæmorrhages from the urethra. Certain diseases of the skin have been described, and quite appropriately, as dermato-neurosis.

Leloir states that certain lesions of the skin are a premonition of threatening neurotic trouble of a serious character. For example, one of his patients suffered with a severe herpes zoster of the right side of the chest for a short time, having been previously healthy. Six months later paraplegia and paralysis of the sphincters occurred as a consequence of syphilitic myelitis. In a second case he observed the existence of a patch of non-parasitic alopecia areata for a short time prior to the development of the cerebral lesion of syphilis. The same lesion in another case apparently heralded the development of the cerebro-spinal syphilis and general paralysis. In still another case labial herpes and scleroderma in circumscribed patches occurred from time to time for several years, and were followed by general paralysis.

Duplay records one case in which zoster of the inferior extremity led to the discovery of Pott's disease of the spine. In another case, recorded by the same observer, œdema of the lower limbs, with pigmentation of the skin, led to a diagnosis of spinal meningitis.

Raymond cites a case in which intense herpes of the pharynx preceded a cerebral lesion. Eczema and neuralgia oftentimes precede the development of chorea.

In my essay upon tropho-neurosis in syphilis I called especial attention to the association of alopecia areata and herpes zoster to neurotic disturbances. My friend, Prof. Ohmann-Dumesnil, of St. Louis, has reported several cases of alopecia due to traumatism.

My attention was first called to the possible dependence of syphilitic lesions upon tropho-neuroses by a series of cases of necroses of the maxilla, alveolar processes, palate and bones of the nose, occurring in cases of tertiary syphilis. In studying these cases I was led to pursue the line of thought a little further and I found that evidences of the dependence of syphilitic phenomena upon organic or functional disturbances of the sympathetic system are quite positively manifested here and there along the whole line of morbid phenomena developed in the course of the disease. There is not a lesion of syphilis which can not, it seems to me, be explained upon this theory. Even the affinity of syphilis for the lymphatic glands appears to be analogous to those phenomena which occur in Hodgkin's disease and leucocythæmia—diseases which are inexplicable save upon the neurotic theory. In the late or sequelar syphilides there is a special tendency to disturbances of a tropho-neurotic character. It would appear that syphilitic infection not only has a peculiar affinity for the sympathetic nervous system, but that this affinity is particularly marked in the upper or cervical portion of the sympathetic. All through the disease the proportion of lesions about the head, face and mouth is relatively much larger, even under the best treatment, than in other portions of the body. The parts supplied by the fifth cranial nerve appear to be particularly susceptible. There are few cases, indeed, no matter how thoroughly they may be treated, that are not affected, at one time or another, with lesions of the lips, inner surface of the cheeks, tongue, throat and scalp. Cases are frequently met with in which the initiatory and active periods of the disease have been passed through without serious trouble, when suddenly and without warning serious destruction of the nasal, palatal or maxillary bones occur.

I have long been impressed by the peculiar course of some of the lesions of late syphilis, particularly those affecting the head, face and oral cavity. It has seemed to me that the destructive effects exerted by the morbid process upon the bony tissue is greatly disproportionate to the objective and subjective phenomena which precede the actual destruction. For example, I think that upon reflection it will be found that the objective morbid phenomena which precede the necrosis *en masse* of various parts of the palate, superior maxillary and nasal bones are comparatively slight, when we take into consideration the fact that the affected bone is entirely destroyed. Indeed, it often seems that the first objective phenomenon perceptible in cases of necrosis of the parts mentioned is incidental, not to destruction of the bone *per se*, but to an attempt on the part of nature to rid the tissues of offending foreign material. Thus I have observed cases in which the greater portion of the palate was entirely destroyed, yet very little manisfestation of trouble was apparent until suppuration occurred, with a small point of ulceration of the soft parts covering the bone, and the discharge of a small quantity of pus—a quantity, by the way, so small as to be entirely disproportionate to the extent of the morbid process. On passing a probe into the small sinus thus formed, one who is not thoroughly conversant with the peculiarities of such conditions will quite likely be surprised to find that a large portion of the bone is dead and perhaps loose in the tissues. It will be found, upon observation of processes other than syphilitic, which produce necrosis or caries of bone, that there exists, prior to the death of the osseous structure, quite pronounced objective phenomena in the way of pain, swelling and deformity of the part, these symptoms indicating the existence of proliferated inflammatory material which subsequently produces, by simple pressure, destruction of the vitality of the bone. Those morbid phenomena in syphilis which involved bone or periosteum in the early part of the course of the disease are accompanied by relatively more prominent objective phenomena than those late lesions which are now under consideration; yet they are rarely followed by caries or necrosis. These processes, it seems, are reserved for the late secondary or sequelar period of the disease. Thus it will be seen that, although the local process is apparently more severe in the early cases, destruction of the vitality of the bone is not so likely to occur. There is a marked difference between the modes and diffuse subperiosteal swellings of early syphilis, and the condition of the bone and periosteum which precedes necrosis *en masse*, or, for that matter, caries in the late stage of the disease. In addition to the disproportion between the degree of destruction of bone and the objective phenomena preceding such destruction, another point worthy of comment is the fact that syphilis possesses the power of dissecting out definite portions of osseus tissue, apparently by cutting off their nutritive supply in a manner as cleanly as it could be done by the knife. Thus, I have specimens in my possession of the intermaxillary bone, portions of the alveolar process of the maxilla, the palatal and nasal processes of the superior maxillary, the molar and ossæ nasi, which became necrosed, loosened and were removed from cases of late syphilis. These fragments of bone present as natural a conformation in many instances as in their healthy condition.

As far as I have been able to observe, there seems to be a special predilection in cases of late syphilis for those parts supplied by the fifth nerve, indicating that the portion of the sympathetic system which presides over these parts is particularly sensitive to the syphilitic impression.

I have found in some instances the tendency to unilateral destruction of osseous tissue particularly marked. Thus, the palatal process of the superior maxilla upon one side, or superior alveolus upon one side, may necrose and give way without the corresponding portion of bone becoming affected. Indeed, it seems that in most instances in which necrosis attacks the bones of the face it is impossible to check the process until the line of demarkation represented by the anatomical outline of the affected bone has been reached. The peculiar manner in which one-half of a structure may be dissected away by the sequelar lesions of syphilis is exemplified by a case of syphiloma of the tongue which recently came under my observation, in which the sloughing of the organ was limited by the raphe. This case subsequently went on to malignant transformation. I removed the tongue by the galvano--cautery, the disease recurred, and the patient died of hæmorrhage several months later.

I have had several cases recently in which that portion of the superior maxilla corresponding to the intermaxillary bone was dissected out by the syphilitic process, with the resultant loss of the incisor teeth, the remainder of the jaw remaining intact. There appears to be a peculiar predilection of late syphilis for this portion of the jaw. I have seen several cases in which caries occurred in this situation, with a consequent loss of one or more perfectly healthy teeth. These cases have appeared to me to be so characteristic that I have come to regard the loss of the incisor teeth without any apparent cause as almost positive evidence of syphilis.

An interesting case illustrating the unilateral limitation of some late lesions of syphilis came under my observation recently. This was a gentleman who had an obscure history of syphilis, dating some years back. Several weeks before coming under my observation ulceration began at the roots of the molar teeth upon one side and extended outward upon the palate. When I first saw the case the ulceration had extended outward upon the hard palate for about three-quarters of an inch, and forward to the median line, where it abruptly stopped. The appearance of the ulceration was quite typical. There was no disease of the teeth or jaws to account for it. Healing was quite rapid under appropriate antisyphilitic treatment.

Another interesting case of a somewhat similar character is that of a gentlemen who had syphilis seven or eight years ago. For the last three or four years he has had occasional symptoms of the disease. A few months since ulceration occurred about the roots of the upper incisor teeth, and was attended with slight caries of the intermaxillary bone. The process was checked by appropriate treatment, the teeth, which are loosened, finally becoming perfectly solid. About six or eight weeks after the ulceration was healed the patient consulted me for supra- and infra-orbital neuralgia and hemicrania. It yielded readily to iodine of potassium in large doses. Within a few days the patient has again consulted me for paræsthesia of the right side of the face, which he noticed for the first time while being shaved. His face had been excessively tender previously, he very speedily noticed a lack of sensibility under the razor. Associated with this paræsthesia there is obscure pain, which he locates back of the eyeball. The *ensemble* of symptoms in this case points to central disturbance, and evidence a manifest predilection of the sequelar lesion for the fifth cranial nerve.

The association of obstinate tubercular syphilides with nervous syphilis is well known. It seems that the danger of involvement of the central nervous system is directly proportionate to that of severe syphilides.

In considering the tropho-neurotic character of the late lesions of syphilis, I do not not ignore the fact that syphilis may act directly upon the nervous system in several different ways :

1. By the direct effect of syphilitic deposit upon the nerve cells or fibres, or membranes of the brain and spinal cord.

2. By changes in the membranous envelopes of the brain and spinal cord.

3. By deposits in and about the blood vessels, which induce circulatory disturbances.

4. By a proliferation and condensation of connective tissue, which remains after the syphilitic material *per se* has been removed.

There is probably a difference in the late and early forms of syphilitic lesion in the manner in which the tropho-neurotic element is brought about. Thus, it may be due in the first instance, to a direct impression of the syphilitic poison upon the sympathetic nervous system; secondly, upon direct pressure upon the nervous structures; thirdly, upon a disturbance of function and nutrition of the nervous structures incidental to interference with blood supply.

It is probable that mercury acts upon the nervous system in very much the same manner as does syphilis. It is very difficult to differentiate late syphilitic lesions of the bones and of the mucous membranes from those directly due to the action of mercury. That mercury exerts a powerful effect upon the sympathetic nervous system is, it seems to me, shown conclusively by the phenomena of ptyalism, which can not be accounted for solely upon the theory of the production of irritation. The well known power of mercury over the secretions is probably due to its influence upon the sympathetic ganglia. When the injurious action of mercury is superadded to syphilis, there is a more marked tendency to tropho-neurotic phenomena than in well treated cases of the disease. Indeed, the excessive use of mercury often seems to determine the predilection of late syphilis for the bones of the head and face. It is quite as capable of producing necrosis or destructive ulceration of these parts as is syphilis *per se*.

Positive demonstration of the dependence of the phenomena which I have outlined upon nervous disturbance is of course difficult, but the inferences which I have drawn appear to me to be logical.

In considering the question of trophic disturbances in their relation to destructive syphilitic processes, it is well to remember the familiar physiological experiment of section of the sympathetic in the neck of the rabbit. The same experiment is also interesting as bearing upon the faucial congestion of early syphilis. The reddening of the ear of the rabbit, the inflammation and sloughing of the cornea incidental to section of the sympathetic, are certainly suggestive. To carry the analogy of this physiological demonstration a little further, I would call attention to the serious corneal trouble which sometimes results from herpes frontalis seu orbicularis.

The tropho-neurotic influence of syphilis appears to be chiefly manifested in the peripheral structures of the body. Thus, in late syphilis we have a tendency to brittleness and other morbid chances of the finger and toe nails. There is falling of the hair, due to intrinsic perversion of vitality of the hair follicle, and differing from the alopecia areata of the early stages of the disease. The most important evidences of the tropho-neurotic influence of syphilis is the malnutrition of the teeth observed in syphilitic children. In my opinion syphilis may impress several generations of individuals with a tendency to tropho-neurotic changes of the glands, teeth, nails, etc., long after syphilis *per se* has been eradicated. It is my opinion that scrofula is frequently the result of this neurotic tendency, *i. e.*, tropho-neurotic disturbance.

In a paper read before this Section at the meeting of the American Medical Association, June 12, 1886, I directed attention to the close similarity which exists between so-called canker of the oral cavity and certain syphilitic lesions. This resemblance I believe to be due to the fact that both are the result of tropho-neurotic disturbances; in the one case produced by syphilis, or syphilis and mercury combined, and in the other to general perversion of nutrition, or, more frequently, disturbances of the digestive apparatus.*

*In a paper read before the North Texas Medical Society, I called attention to the relation of herpes progenitalis upon the disturbed enervation incidental to syphilis. (Philadelphia Medical News, Feb. 8, 1890.)

Dr. Hadden‡ reports four cases which I believe to be due to tropho-neurotis disturbance. These cases were absolutely resistant to treatment, and consisted of a sensation of intense unbearable burning of the tongue and often the lips and roof of the mouth. In two of these cases there were certain objective symptoms. In one, a woman of 35, there was a small epulis; in another, a woman of 75, who was emotional and nervous, and had for many years suffered from nettle rash, the gums finally became involved and the teeth turned black and decayed.

